# Somato-Motor Haptic Processing in Posterior Inner Perisylvian Region (SII/pIC) of the Macaque Monkey

**DOI:** 10.1371/journal.pone.0069931

**Published:** 2013-07-30

**Authors:** Hiroaki Ishida, Luca Fornia, Laura Clara Grandi, Maria Alessandra Umiltà, Vittorio Gallese

**Affiliations:** 1 Italian Institute of Technology (IIT), Brain Center for Motor and Social Cognition (BCSMC), Parma, Italy; 2 Department of Neuroscience, Unit of Physiology, Parma University Parma, Italy; University of Ottawa, Canada

## Abstract

The posterior inner perisylvian region including the secondary somatosensory cortex (area SII) and the adjacent region of posterior insular cortex (pIC) has been implicated in haptic processing by integrating somato-motor information during hand-manipulation, both in humans and in non-human primates. However, motor-related properties during hand-manipulation are still largely unknown. To investigate a motor-related activity in the hand region of SII/pIC, two macaque monkeys were trained to perform a hand-manipulation task, requiring 3 different grip types (precision grip, finger exploration, side grip) both in light and in dark conditions. Our results showed that 70% (*n* = 33/48) of task related neurons within SII/pIC were only activated during monkeys’ active hand-manipulation. Of those 33 neurons, 15 (45%) began to discharge before hand-target contact, while the remaining neurons were tonically active after contact. Thirty-percent (*n* = 15/48) of studied neurons responded to both passive somatosensory stimulation and to the motor task. A consistent percentage of task-related neurons in SII/pIC was selectively activated during finger exploration (FE) and precision grasping (PG) execution, suggesting they play a pivotal role in control skilled finger movements. Furthermore, hand-manipulation-related neurons also responded when visual feedback was absent in the dark. Altogether, our results suggest that somato-motor neurons in SII/pIC likely contribute to haptic processing from the initial to the final phase of grasping and object manipulation. Such motor-related activity could also provide the somato-motor binding principle enabling the translation of diachronic somatosensory inputs into a coherent image of the explored object.

## Introduction

Whenever retrieving a key or lipstick from the bottom of a purse, we usually identify the searched object by hand-finger exploration in the absence of vision [1]–[4]. Despite our proficiency at haptic perception and our reliance on it in every day life, the neuronal mechanism behind this somato-motor process remains largely unclear.

In both humans and non-human primates, the secondary somatosensory cortex (area SII) and the adjacent posterior insular cortex (pIC) is believed to play a pivotal role in high-level haptic perception [5]–[8]. Neuropsychological studies revealed that unilateral damage to parieto-temporal cortices in either hemisphere, possibly including SII, induces tactile agnosia [9] and tactile apraxia [10]. In fact, both types of patients exhibit abnormal hand-manipulation [11], frequently accompanied by impairments of tactile object recognition in the absence of more basic somesthetic dysfunction [12], [13]. Furthermore, the degree of recovery of manual dexterity in stroke patients revealed that it more positively correlates with the activation of SII than of the primary somatosensory cortex (SI) [14]. This evidence suggests that fine manual control and haptic perception closely tie to each other in area SII. In favor of this interpretation, neuroimaging studies also demonstrate that human SII and ventral premotor cortex (PMv) are more activated during active finger movements than during passive ones [15]. Moreover, the activation of SII-PMv is particularly strong during hand-manipulation tasks in which complex object manipulation was compared to simple object manipulation ones [16].

In non-human primates, area SII and adjacent pIC show multiple digits and hand representations [17]–[20]. Animal lesion studies also demonstrated that the unilateral ablation of SII in monkeys produced severe impairments both in texture and shape discrimination learning [21], [22]. Concerning a hand-manipulation-related neuronal network in macaque monkey brain, the hand regions within SII/pIC are characterized by the presence of reciprocal connections with the parieto-premotor hand-manipulation-related areas, such as ventral premotor area F5 and anterior intraparietal area AIP [23]–[27]. Single neuron recording [28]–[34] and fMRI studies [35], [36] in monkeys demonstrated that the parieto-premotor areas play crucial roles in the visuo-motor transformation necessary for grasping objects. The visuo-motor model for object grasping suggests that area AIP sends visual information of objects to area F5 for selection of the pattern of hand movement, and area F5 sends back the motor signal (efference copy) of the selected motor command to area AIP [37]–[39]. Given the above mentioned arguments, there are at least two questions to be raised: 1) Are SII/pIC neurons involved in sensory guidance of voluntary movement in addition to their role in somatosensory perception?; 2) Whether, and to what extent, does visual feedback affect their neuronal activity?

**Figure 1:**
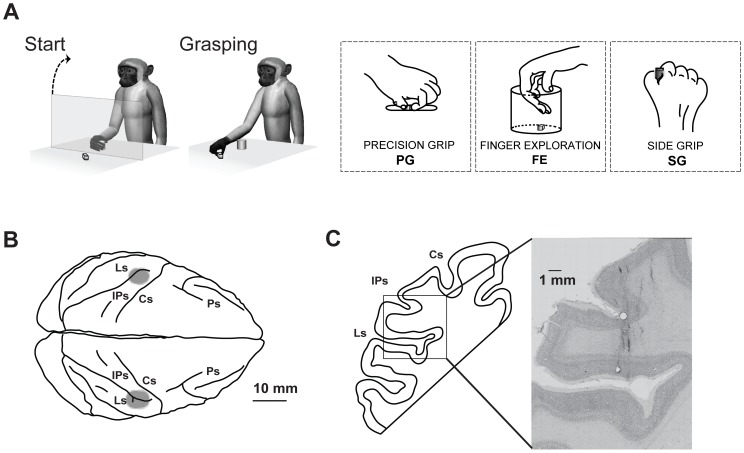
Recording sites and motor task. (*A*) Motor task. The monkey started the task with the hand in a fixed resting position. A rigid transparent screen was interposed between monkey’s hand and target. When the screen was removed (Start), the monkey reached for and grasped the target (Grasping). Grip types employed in the motor task. (*B*) Top view of the brain of monkey 1 (MK1). Gray shaded regions indicate estimated entrance points of microelectrodes from the convexity in both hemispheres. Ps, principal sulcus; CS, central sulcus; IPs, intraparietal sulcus; Ls, lateral sulcus. Calibration bar: 10 mm. (*C*) Reconstructed coronal section and enlarged Nissl microphotograph of the same section showing microelectrode tracks. Calibration bar: 1 mm.

In order to answer these questions we investigated hand-manipulation-related neurons in SII/pIC in monkeys trained to perform a hand-manipulation motor task, using three different grip types (retrieving a food morsel from a groove, a cup, a plate; Figure 1A). We hypothesized that activation of SII/pIC neurons during hand-manipulation would reflect either the direct/indirect influence of selected motor signals or the predicted sensory consequences of motor command, likely in virtue of the reciprocal connections between area F5 and SII. In terms of visual responses, we expected that neurons in this area might be involved in directing somato-motor attention during hand-manipulation in the absence of vision [40]–[47].

Our results show that a subset of neurons within SII/pIC hand region is only activated during monkeys’ active hand-manipulation. These neurons were selectively activated during finger exploration (FE) and precision grasping (PG) execution. Furthermore, a subset number of hand-manipulation-related neurons increased their discharge in the dark. The temporal profile of task-related responses enabled us to segregate three possible components of haptic processing in SII/pIC, namely: 1) Prediction of the hand-finger movement; 2) Hand-object contact detection; 3) Hand-finger exploration. Such somato-motor haptic processing could also provide the somato-motor binding principle enabling the translation of diachronic somatosensory inputs into a coherent image of the explored object.

## Materials and Methods

Two male macaque monkeys (*Macaca mulatta*) were used in this study. We recorded from both hemispheres in one monkey (MK1, 8.0 kg) and from the left hemisphere in another monkey (MK2, 3.5 kg). All experimental protocols were approved by the Ethical Committee for Animal Research of the University of Parma and by the Superior Institute for Health (last appraisal no. 2783, 26/01/2010). The authorization for conducting our experiments was delivered by the Animal Health and Veterinary Medication Division of the Department of Public Veterinary Health, Nutrition and Food Safety of the Italian Ministry of Health (permit by ministerial decree no. 6/99-A, 29/01/1999; last renewals. no. 54/2010-B, 55/2010-C, 18/03/2010). The monkeys were housed and handled in strict accordance with the recommendations of the Weatherall Report about good animal practice. For example, the monkeys were fed a variety of vegetables, fruits, and grains everyday. Supplementary pellets were also provided for maintaining their nutritional health. Their health condition (e.g., body weight, behavior and appetite) was carefully checked by experimenters everyday. The monkeys were kept in individual primate cages (Tecniplast S.p.A, Bugugiate, Italy, Approximately 180 cm height, 90 cm wide, 120 cm depth) in an air-conditioned room where was maintained a consistent temperature at approximately 25–26 degrees Celsius. Our routine laboratory procedures also included an environmental enrichment program where monkeys had access to toys, mirrors and swings. They also had visual, auditory and olfactory contact with other animals and, they could touch/groom each other. Any possible pain associated with surgeries was pharmacologically ameliorated. The well-being and health conditions of the monkeys were constantly monitored by the institutional veterinary doctor of the University of Parma.

### Task Training and Surgical Procedures

Before recordings, each monkey was habituated to comfortably sit in a primate chair, to interact with experimenters and to become familiarized with the experimental setup. Then, they were trained to perform the motor task described below using the hand contralateral to the hemisphere to be recorded.

At the end of training of MK1, a head fixation system and custom-made rectangle-recording chambers (inner dimensions 30 mm×15 mm) were implanted over both hemispheres based on stereotaxic coordinates of the cortical regions to be recorded (Figure 1A, B). The surgery was performed under general anesthesia (ketamine hydrocloride, 5 mg/Kg, i.m. and medetomidine hydrocloride 0.1 mg/Kg i.m.), followed by post-surgical pain medications. Surgical procedures were the same as previously described [48].

In the case of MK2, the targeted area was identified on MRI images prior to the experiment. The first surgery was performed to enable head fixation under the above-described anesthesia. A “K-letter” shaped stainless steel head post (Crist Instrument, Hagerstown MD, USA) was implanted on the occipital skull to allow attachment of a head-fixation bar on the primate chair. Following training, a cylindrical-recording chamber (Narishige, Tokyo, Japan, inner diameter = 20 mm) was implanted under above-described anesthesia.

The center of the chamber and angle in stereotaxic coordinates were as follows: in MK1 over both hemispheres (anterior [A] = 11.0 mm; lateral [L] = 20.0 mm; angle 45°) and in MK2 over the right hemisphere (A = 13.0 mm; lateral L = 15.0 mm; angle 90°). The position of all chambers allowed recording from the rostral to the middle part of the upper bank of the lateral sulcus, including the hand regions of SII/pIC (see Figure 1 B, C and Figure S1).

### Recording Procedure and Recording Sites

Single-unit recording was performed extracellularly using varnish-insulated tungsten microelectrodes (impedance 0.5–1.5 MΩ at 1 kHz; FHC, USA) advanced perpendicularly into the cortex through the dura matter. In the MK1, the terminal of hydraulic microdrive manipulator (TrentWell, CA, USA) was attached to a stereotaxic arm and fixed to the monkey’s head fixation apparatus on the monkey’s chair. In the MK2, the microelectrode was mounted on an electrode-driving terminal (MO-97, Narishige, Tokyo, Japan) fixed onto the recording chamber. Neuronal activity was amplified (Model A–I, BAK Electronics, Germantwon MD, USA) and monitored on an oscilloscope. Single neuron action potentials were isolated on-line with a dual voltage-time window discriminator (Model DIS-I, BAK, Electronics, Germantwon MD, USA) to test properties of single neurons. Raw analog signal, isolated action potentials and the digital events related to the behavioral paradigm, were acquired and stored on-line by means of CED1401 mk-II and Spike2 software (Cambridge Electronic Design, Cambridge, UK). A waveform of single spike was further extracted and sorted off-line using the same software.

After recording chamber implantation, physiological boundaries of hand regions in area SII/pIC were identified on the basis of stereotaxic coordinates and previously described neuronal properties (Figure 1B, C and see also Figure S1) [17]–[19], [26], [49]. In accordance with previous studies in the posterior inner perisylvian region including SII/pIC, we found the hand and arm representations in the middle part of the upper bank. We also found that the face and oral structures (teeth, gums, palate) were represented in the rostral part, while the foot and leg were represented in the caudal part. Since the present study focused on purely motor responses of SII/pIC neurons, we only recorded neurons if we clinically observed stronger responses during active hand-manipulation than during passive somatosensory stimulations.

### Behavioral Testing and Apparatus

#### Somatosensory stimulations

Passive somatosensory stimuli consisted of a) ‘superficial tactile’ stimulation (T), consisting of hair deflection by touch or light pressure to stimulate subcutaneous tissues; b) ‘proprioception’ (P), consisting of slow and fast passive joints movement of the upper limb (the shoulder, elbow and wrist) and fingers phalanxes. Neuronal activity was recorded while passive somatosensory stimuli were applied to monkeys’ body parts by using experimenter’s hand in the absence of any visual feedback [48], [50]. If we found superficial tactile RFs, we applied a stimulus to the body part. If we found proprioceptive dominant responses, we manipulated the joint (ex. flexion or extension).

#### Hand-manipulation task

Monkeys were trained to perform a modified version of a motor task previously described [51]. Figure 1A shows the motor task employed in this study. In order to study possible grip-selectivity, monkeys were trained to perform the motor task using three different grip types. Monkeys were trained to perform a) ‘precision grasping’ (PG): by using the pulpar surface of the distal phalanxes of thumb and of the index finger, when a small piece of food (a cube of 1 cm size) had to be grasped from a groove; b) ‘finger exploration’ (FE): by using the second and third digits working together in opposition to the thenar eminence, when a small piece of food had to be taken out from a cup (inner diameter 3 cm and depth 3 cm); c) ‘side grasping’ (SG): by using the distal pad of the thumb opposed the radial surface of the distal phalanx of the index finger, when a small food had to be picked up from a plate. Each trial started with a set period for 2–3 sec during which monkeys were holding a home key. During such period, a transparent plastic screen was interposed between the monkeys’ hand and the target. When the screen was removed (go signal), monkeys released the key and grasped the target employing one of the three grip types and ate the food (Figure S2).

#### Hand-manipulation in the dark

In order to examine the influence of visual feedback during hand-manipulations, monkeys were trained to perform the task in the dark. In this condition, the target was briefly illuminated (1 sec) then light was turned off before go signal. We trained monkeys to perform the task and to recognize the go signal even in the dark. Since the go signal was the removal of the screen placed close to the monkeys’ face, it could be easily detected in the dark.

The average duration for the reaching and pre-shaping hand/finger movement before the hand-target contact was 402±66 msec in Light and 513±113 msec in Dark condition. The mean duration of hand-manipulation after the hand-target contact was 264±145 msec in Light and 472±260 msec in Dark condition. Finally, the mean duration of the bringing to mouth movement was 343±79 msec in Light and 379±114 msec in Dark condition (for each index both in Light and Dark conditions, *n = *108 trials includes three different grips; for detailed information, see Figure S2).

#### Effect of target presence

To examine the role of target presence during FE execution, MK2 performed two FE tasks in the dark: a) FE task with a target; b) FE task without a target (Figure S3). Both FE and FEwt tasks were administered in a pseudorandom fashion. Since the monkey could not see the target inside a cup, its presence could not be anticipated until the monkey moved its fingers inside it. In FE task, the monkey took a small piece of food from a cup and ate it. The mean duration of hand-manipulation execution and bringing to the mouth time was 548±170 and 522±144 msec respectively. In FEwt task, the monkey explored inside the cup and once he understood the absence of the target, he returned its hand onto the home key. At this moment, trial ended. The mean duration of hand-manipulation execution and returning the hand to the key was 723±100 and 422±122 msec respectively. If monkey capture searching inside the cup for more than 1 sec, trial was discarded and not included in the data.

Both somatosensory stimulations and hand-manipulation tasks were administered in a pseudorandom fashion. Trials were discarded and not included in the data set, if monkeys: a) moved their body parts during passive somatosensory stimulations; b) lifted their hand from the home key before the go signal; c) failed to correctly grasp the target. In hand-manipulation tasks, a contact detecting electric device was used to signal the contact of monkey’s hand with the target in devices for each motor task. In FE/FEwt tasks, the device signaled the contact of monkey’s hand with bottom of the cup. During somatosensory stimulations, a foot switch was used to signal the somatosensory event. Both digital signals were used for subsequent alignment of neuronal activity and for statistical analysis of neuronal discharge in different epochs. In order to calculate mean duration of both somatosensory stimulations and motor tasks, we complementarily recorded them by means of a digital video camera (25 frames per second). Videos were analyzed frame-by-frame by means of homemade dedicated software off-line. The mean duration of tactile and proprioceptive stimulation was 341±78 and 389±160 msec respectively.

### Data Collection and Analyses

#### Task epochs

Neuronal activity was recorded from 2 seconds before until 2 seconds after the somatosensory event onset or hand-target contact (4 seconds for each trial). We subdivided hand-manipulation-related responses into 3 epochs: 1) Baseline activity, starting 2 seconds before the hand-target contact and lasting for 400 msec, when the hand was at rest on the starting position; 2) Pre-contact, lasting 400 msec before the hand-target contact; 3) Post-contact, lasting 400 msec after the hand-target contact. In the somatosensory responses, each trial was subdivided into two epochs: 1) Baseline activity, starting 2 seconds before the somatosensory stimulation and lasting for 400 msec; 2) Somatosensory stimulation, the mean duration of somatosensory stimulation was approximately 400 msec.

The mean discharge frequency during the above-defined stimulation epochs for each somatosensory stimulation and motor task was compared with mean activity during baseline by means of a Wilcoxon test and a 3×3 repeated measures ANOVA (factors: Grip, Epoch) by following Bonferroni correction, respectively. All the neurons presented in this study displayed statistically significant responses during the above-defined grasping or somatosensory stimulation related epochs with respect to baseline. All analyses were performed using a significance criterion of *p*<0.05. Statistical analyses were performed using Matlab (The MathWorks Inc., MA, USA) and Statistica software (StatSoft). Subsequently, if a neuron showed significant responses during passive somatosensory stimulations, we assigned it to the category ‘somatosensory (SS)-related’ neuron. These neurons were analyzed separately from hand-manipulation-related ones.

#### Grip-preference

To quantify the preference of recorded single neurons for the different grip types, we calculated a preference index (PI) taking into account the magnitude of the neuronal response to the three grips. It was calculated as follows:
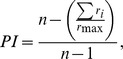
where *n* is the number of grips (*n* = 3), *r_i_* is the mean firing rate of the neuron in its pre- and post-contact epoch of each grip and *r*_max_ is the maximal mean value for the preferred grip during its pre- and post-contact epochs. PI values can range from 0 (the discharge is identical among grips) to 1 (maximal selectivity for one grip).

#### Population analysis

Population response was calculated as a net normalized mean activity. First, the mean activity was calculated for each 20 msec bin through all the recording trials of each condition. Then, an offset procedure was applied for each condition, subtracting the mean baseline activity from the value of each bin (net activity). For each neuron, the peak discharge was found over all task conditions during task-related epochs and used to normalize activity of each condition. To statistically compare responses in different populations, we used the net normalized mean activity as a dependent variable. We then performed a 3×2 repeated measures ANOVA (factor: Grip, Epoch) followed by Bonferroni post-hoc tests (*p*<.05*).*

#### Histological reconstruction and identification of the recorded regions

About 1 week before sacrificing the monkey (MK1), electrolytic lesions (10 µA cathodic pulses, duration 10 s) were performed at known coordinates at the external borders of the recorded regions. The monkey was then deeply anesthetized and perfused as previously described [52]. The brain was then extracted, photographed, and cut (slice thickness 60 µm). Each second and fifth section of a series was stained using the Nissl method (thionin, 0.1% in 0.1 M acetate buffer, pH 3.7). The locations of penetrations were then reconstructed on the basis of electrolytic lesions, stereotaxic coordinates, depths and functional properties of each penetration.

## Results

We recorded a total of 277 single units from the posterior inner perisylvian region, including area SII and the adjacent region of posterior insular cortex (pIC) of two macaque monkeys (179 in the two hemispheres of MK1, 98 in one hemisphere of MK2). Figure 1B shows the anatomical location of the investigated regions in MK1, and Figure 1C shows an example of Nissl section with the recording tracks, in the left hemisphere of the same monkey.

On the basis of the result of our clinical test, 151 out of 277 neurons (55%) were categorized as hand-related-somatosensory or motor neurons. One-hundred-eleven out of 151 neurons (74%) showed clear somatosensory receptive fields (RFs) on the hand or fingers. The remaining 40 neurons (26%) did not show any responses during passive somatosensory stimulations, thus making it impossible to identify the location of either tactile or proprioceptive RFs on the hand and fingers. These neurons responded specifically during hand-manipulations performed during the motor task (Figure 1A). Thirty-five out of 277 neurons (13%) were mouth-related somato-motor neurons. Of these, 20 neurons showed responses during active mouth movements. The remaining neurons (*n* = 15) showed clear somatosensory RFs on the external skin around the mouth or on intraoral structures.

Concerning other passive somatosensory representations, 24 out of 277 neurons (9%) showed RFs on the upper arm. The remaining neurons showed RFs on the lower body (*n* = 21, 7%), face (*n* = 13, 4%) and upper body parts (*n* = 6, 2%). Finally, twenty-seven out of 277 neurons (10%) either showed audio-visual or somato-visual multimodal responses or they did not respond to the motor task.

Within the above-described 190 somatosensory dominant neurons with clear RFs, 80% of them responded to light tactile stimulation, while the remaining showed proprioceptive- and joint-related responses. Of these 190 neurons, most of their RFs were located on the contralateral side of the body (62%), while some had RFs bilateral or centrally (30%) or the whole body or the hemibody (8%).

### Hand-related Somatosensory and Motor Responses in SII/pIC

Forty-eight out of 151 hand-related somato-motor neurons could be quantitatively studied in all experimental conditions. On the basis of the results of single units statistical analysis, two main classes of neurons were differentiated: (1) Thirty-three (68%) exhibited statistically significant hand-manipulation-related activity in the pre- and/or post-contact epochs but did not show any significant somatosensory-related responses (T or P; for both responses, *p*>.05, *n.s.*). They were classified as hand-manipulation-related neurons. (2) Fifteen neurons (32%) were classified as somatosensory (SS)-related neurons since their responses during tactile (T) and/or proprioceptive (P) passive stimulations were significantly stronger than during baseline (*p*<.05). They showed somatosensory RFs on the contralateral side of the thumb (*n* = 4), index finger (*n* = 3), multiple-fingers (*n* = 4), palm (*n* = 2) or on the bilateral thumb (*n* = 1) or hand (*n* = 1).

### Hand-manipulation-related Neurons

**Figure 2:**
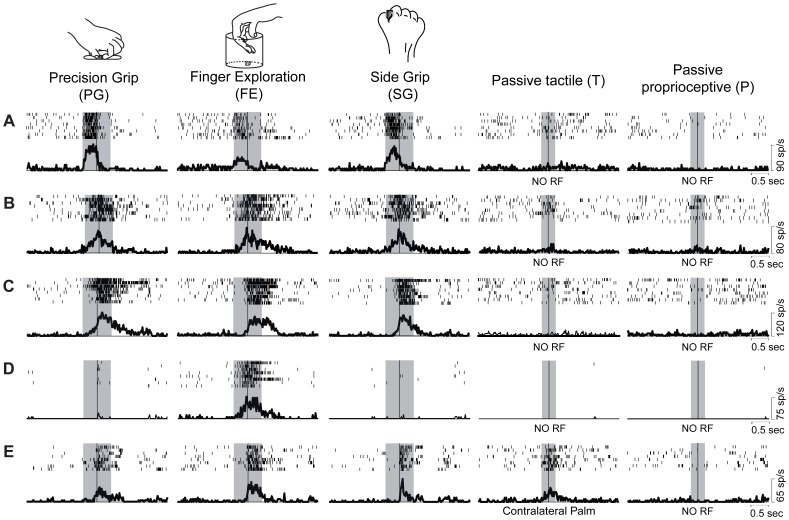
Examples of hand-manipulation-related neurons (A-D) and an example of somatosensory-related neuron (E). (*A*) Example of ‘Pre-contact-selective’ type. This type of neurons shows a vigorous discharge before the hand-target contact in different grip types. (*B*) Example of ‘Middle’ type. This type of neurons discharges before the contact and during holding phase after hand-target contact. (*C*) Example of ‘Post-contact-selective’ type. This type of neurons shows the best response during holding phase. (*D*) Example of a high grip-selective neuron with an high preference index (PI = 1.0). In all hand-manipulation-related neurons (*A-D*), activity during somatosensory stimulation does not significantly differ from baseline activity (*p*>.05, *n.s.,* for both tactile and proprioceptive stimuli). (*E*) Example of somatosensory-related neuron. This type of neurons shows somatosensory receptive field (RF) on the monkey’s fingers and hand with responses significantly stronger than baseline activity (*p*<.05). This type of neurons shows peak discharge at the moment of hand-target contact independently from grip types. Raster and histograms are aligned at the monkey’s hand-target contact. In the somatosensory tests, raster and histograms are aligned at the moment in which the experimenter applied the stimuli onto monkeys’ hands. The gray shaded regions indicate the two hand-manipulation epochs (pre-contact and post-contact). Both in passive tactile and proprioceptive somatosensory stimulations, gray shaded regions indicate a single stimulation epoch. Each epoch lasts 400 msec.

Figures 2A, B, C and D show 4 representative examples of hand-manipulation-related neurons. All of them did not show any significant response to passive somatosensory stimulations.

**Figure 3:**
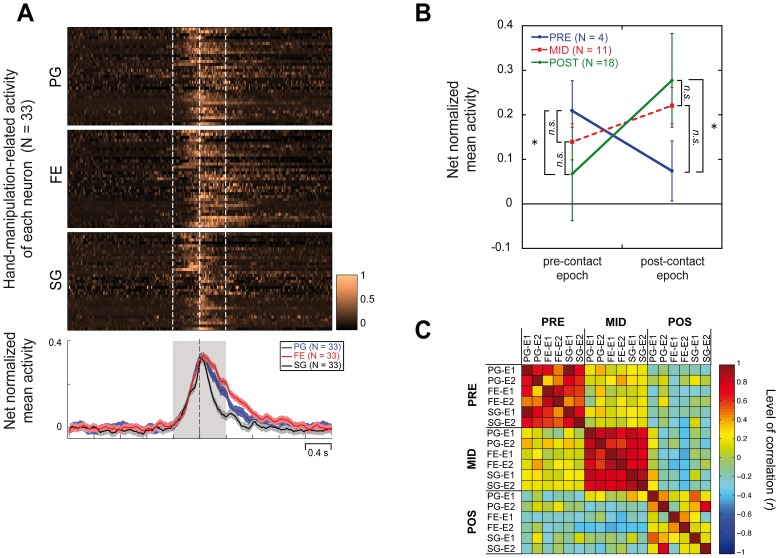
Population analysis of hand-manipulation-related neurons. (*A*) Net normalized activity of 33 neurons for each grip (upper illustration). Temporal profile of the mean normalized activity of the whole neuronal population for each grip (bottom illustration). Raster and histograms are aligned at the monkey’s hand-target contact. The gray shaded regions and the area between white dashed lines indicate the two hand-manipulation epochs (pre-contact and post-contact). (*B*) Mean net normalized response in Pre-contact, Middle and Post-contact-selective type of neurons in the pre- and post-contact epochs. Bars indicate ± SEM, *p<.01. (*C*) Correlation analysis among mean net normalized responses to each of the 3 grips (PG, FE and SG) and 2 epochs (E1, E2) in the 3 types (PRE, MID and POS). Color code indicates correlation value (*r*) between all possible pair of responses.

Since single unit analysis demonstrated a significant difference between pre- and post-contact activity in hand-manipulation-related neurons (Figure 3A upper panels), we further investigated the temporal profile of their discharge. We statistically subdivided hand-manipulation-related neurons into 3 types: 1) Pre-contact-selective (PRE, *n* = 4/33), which showed significantly stronger responses in pre-contact epoch than in post-contact one (ex. Figure 2A); 2) Post-contact-selective (POS, *n* = 18/33), which showed significantly stronger responses in the post-contact than in pre-contact epoch (ex. Figure 2C); 3) Middle-type (MID, *n* = 11/33), which did not show significant difference between the two epochs (ex. Figure 2B).

Figure 2A shows an example of Pre-contact-selective neurons. The activity was significantly stronger in the pre-contact epoch than in the post-contact one [F(2,14) = 228.6, *p*<.0001]. Moreover, the temporal pattern of discharge was similar for the three tested grips but its firing rate being higher during the execution of PG and SG than during the FE execution [F(2,14) = 8.16, *p*<.001, following Bonferroni correction, for each comparison *p*<.001]. Figure 2 B shows an example of Middle-type neurons, which started to significantly discharge before the hand-target contact and kept firing after hand-target contact. In this neuron, activity both in pre- and post- contact epochs was stronger than during baseline [F(2,14) = 31.7, for each comparison *p*<.001], but neither epochs showed any significant difference between each other (*p* = 0.08, *n.s.*). Moreover, this neuron did not show any grip-selectivity [F(2,14) = 0.1, *n.s.*]. Figure 2C shows an example of Post-contact-selective neuron, whose activity in the post-contact epoch was significantly stronger than during pre-contact one [F(2,14) = 104.6, *p*<.00001]; it was broadly tuned by grasp execution [F(2,14) = 1.9, *n.s.*]. Also Figure 2D shows a post-contact-selective neuron, whose post-contact activity was significantly stronger than pre-contact one [F(2,14) = 22.65, *p*<.0001]. In contrast to neuron of Figure 2C, it showed strong grip-selectivity for FE. Activity during FE execution was significantly stronger than during the execution of the other grips [F(2,14) = 111.05, *p*<.0001, following Bonferroni correction, for each *p*<.001].

To clarify both grip-selectivity and temporal profile discharge of these types of neurons, a 2×3×3 repeated measure ANOVA with Epoch (pre-contact, post-contact), Grip (PG, FE, SG) and Type (PRE, MID, POS) as factors was applied. The analysis did not show any significant main effect but it showed significant interactions both between Epoch and Type [F(2,30) = 34.77, *p*<.0001] and between Grip and Epoch [F(2,60) = 4.68, *p*<.01]. Figure 3B shows the result of the interaction between Type and Epoch. As mentioned above, PRE-type neurons showed significantly stronger activity in ‘pre-contact epoch’ (*p*<.01), POS-type neurons showed significantly stronger activity in ‘post-contact epoch’ (*p*<.01) and MID-type neurons did not showed any significant difference between the two epochs. Moreover, just MID-type neurons did not show any significant interaction with types.

Most importantly, concerning the interaction between Grip and Epoch, activity in the ‘post-contact epoch’ of all 33 neurons was significantly stronger during PG and FE than during SG execution (for both comparisons, *p*<.001), while no difference was present during ‘pre-contact epoch’ (Figure 3A bottom panel).

In order to shed further light onto the relation between hand-manipulation-related activity and the discharge temporal profile of the three neuronal populations (PRE, MID, POS), we calculated the mean values of net normalized activity of each epoch (pre- and post-contact epoch) for each grip (PG, FE and SG) and evaluate their coefficient of correlation (Figure 3C). We found strong positive correlations between each possible pairs of cell within PRE- and MID-type neurons but not within POST-type ones. Among the three different types of neurons, no statistically significant correlations were found (for all cases, *p*>. 05, *n.s.*).

### Grip-selectivity of hand-manipulation-related Neurons

**Figure 4:**
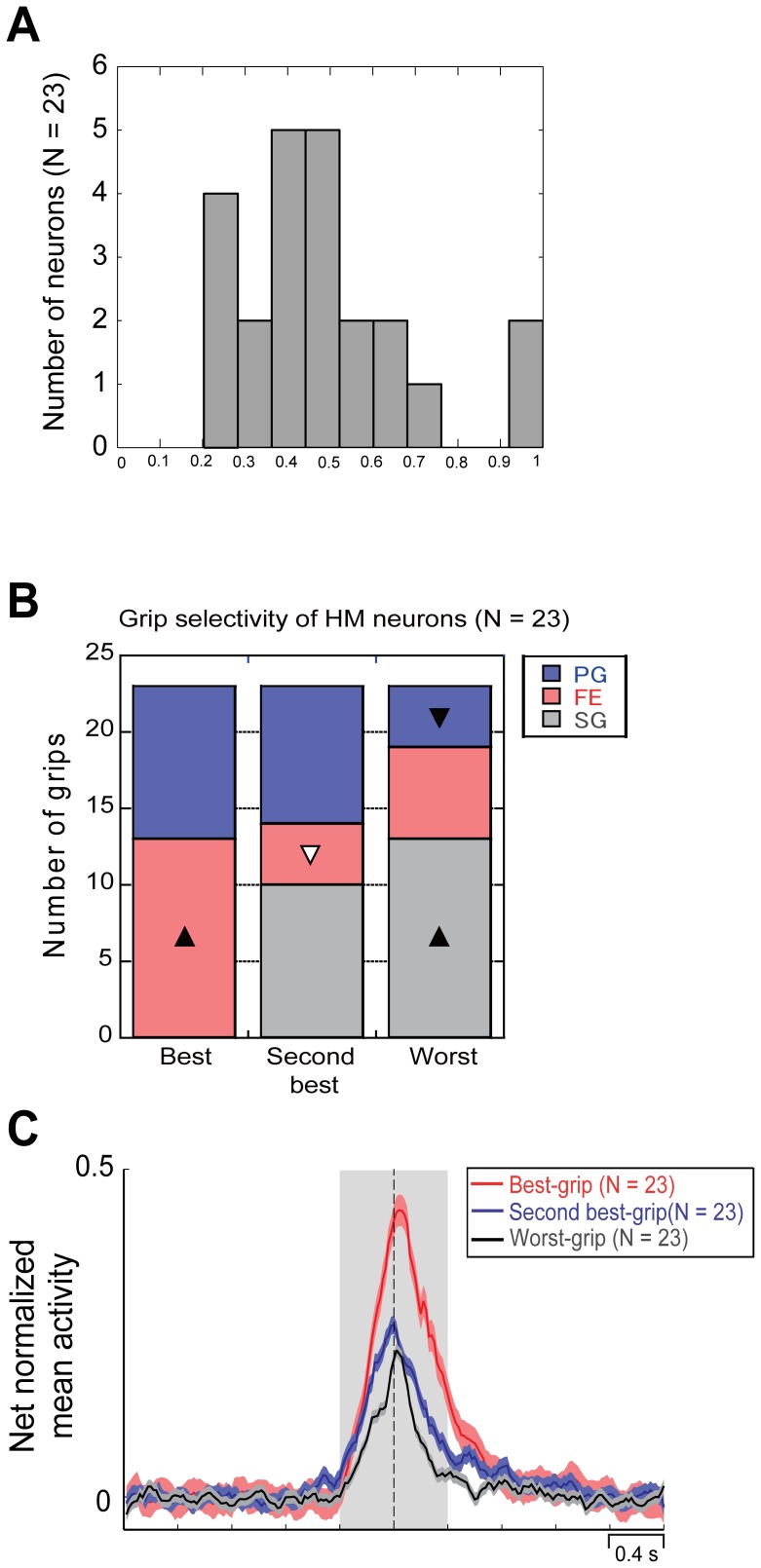
Grip-selectivity of hand-manipulation related neurons. (*A*) Distribution of grip-preference index values. (*B*) Proportion of each grip in three grip selective categories (Best-, Second best- and Worst-grip). Triangles (▴ or △) indicate that the actual measurement of grip is significantly larger than expected values. Inverse triangles (▾ or ▽) indicate the actual measurement of grip is significantly smaller than expected values. Colors of triangles correspond to level of α, namely black corresponds to *p*<.01, white corresponds to *p*<.05. (*C*) Temporal profile of the net normalized mean activity of each grip categories. Conventions as in Figure3.

Since population analysis showed significant main effect for grips (Figure 3A), we further investigated grip-selectivity by focusing on grip-preference of hand-manipulation-related neurons. First, we calculated the grip-preference index (PI, see Materials and Methods) of each single neuron showing a significant main effect for the factor Grip. Since 10 out of 33 neurons did not show any significant main effect for the factor Grip, we excluded them from further analysis. Figure 4A shows the distribution of PI values of grip selective hand-manipulation-related neurons (*n* = 23). For the latter 23 neurons, we compared the net normalized activity mean value of each 3 grips. We then assigned each value into 3 different categories (Best-, Second best- or Worst-grip) in descending order, to evaluate how each grip is represented in each category. Figure 4B shows numbers of each grip in each category. The results of chi-square test in the cross tabulation (3 Grip×3 Category) showed a significant deviation between expected values and actual measurements (*χ* ^2^ = 20.6, *df = *4, *p*<.001). Furthermore, a residuals analysis showed that the proportion of FE in Best-grip (*n* = 13) was significantly the largest (adjusted residual = 2.9, *p*<.01), while that of SG in the same category (*n = *0) was significantly the smallest (adjusted residual = −4.2, *p*<.01). In Second best-grip category, the proportion of FE (*n* = 4) was significantly the smallest (adjusted residual = −2.0, *p*<.05). Finally, in Worst-grip category, the proportion of PG (*n* = 4) was significantly the smallest (adjusted residual = −2.0, *p*<.05), while that of SG (*n* = 13) was significantly the largest (adjusted residual = 2.9, *p*<.01). Figure 4C shows time course and intensity of the activity of neuronal population of each category. To clarify difference among these 3 populations, a 2×3 repeated measures ANOVA with Epoch (pre-contact, post-contact) and Population (Best, Second best, Worst) as factors was applied. The analysis showed a significant main effect for Population [F(2,44) = 61.24, *p*<.0001] but not for Epoch (*p = *0.06). Furthermore, it also showed a significant interaction between factors [F(2,44) = 8.55, *p*<.001]. Activity of Best-grip population was significantly stronger in post-contact epoch than in pre-contact one (*p*<.0001), while Second best- and Worst-grip populations did not show any significant difference between epochs. Furthermore, the pre-contact epoch activity of both Best- and Second best-grip populations was significantly stronger than that of the Worst-grip population (*p*<.0001 and *p*<.05, respectively). In the post-contact epoch, Best-grip population showed significantly stronger activity than both Second best- and Worst-grip population (for both comparisons, *p*<.0001).

### Response Properties of Somatosensory-related Neurons

Figure 2E shows an example of SS-related neuron. The response during passive tactile stimulation was significantly stronger than baseline activity (Wilcoxon matched-paired signed rank test, *p* = .017). This neuron showed tactile RF on the contralateral palm. During the execution of the motor task, it was maximally activated during post-contact epoch and it showed significantly stronger responses during FE than during PG execution (*p*<.0001). This response likely reflected somatosensory stimulation evoked when the fingers but not the thumb touched the palm, a hand configuration typically occurring during FE and SG execution.

**Figure 5:**
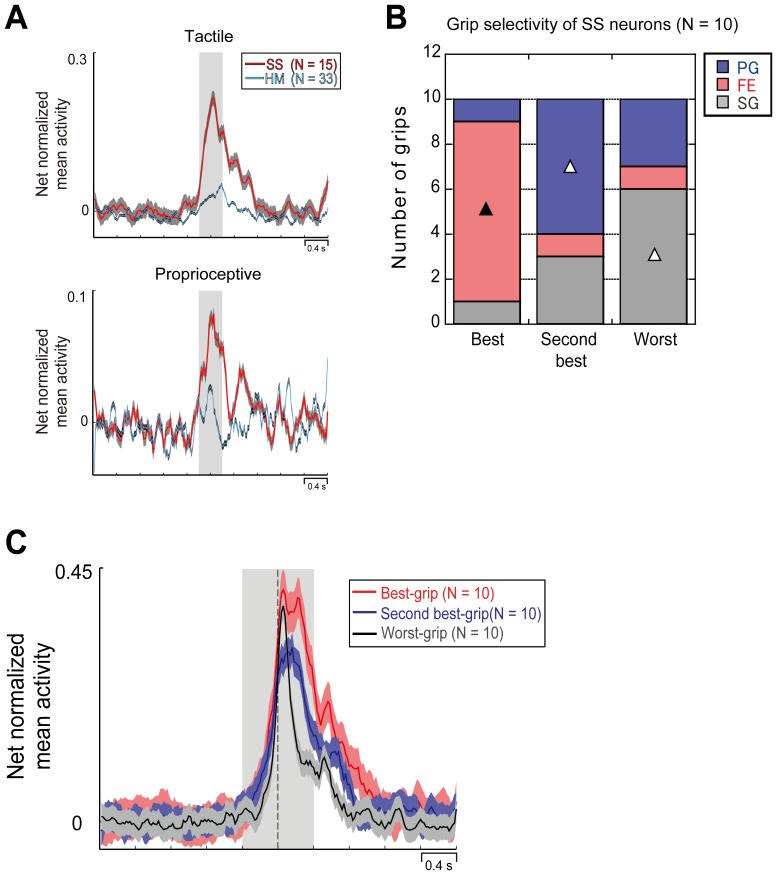
Grip-selectivity of SS-related neurons. (*A*) Temporal profile of tactile and proprioceptive net normalized mean activity of the somatosensory (SS)-related and hand-manipulation (HM)-related populations. Gray-shaded regions indicate stimulation epoch (400 msec). Activity during the somatosensory stimulation was significantly stronger in SS-related population than hand-manipulation-related one (for each, *p*<.0001). (*B*) Proportion of each grip in the three grip categories (Best-, Second best- and Worst-grip). Conventions as in Figure 4. (*C*) Temporal profile of the net normalized mean activity of each grip categories. Conventions as in Figure3.

Figure 5A shows somatosensory responses in the two populations of hand-manipulation-related and SS-related neurons. Although both populations did not show any difference in the baseline epoch, only the SS population showed significant responses during the somatosensory stimulation in the relative epoch (for each, *p*<.0001).

To clarify differences between the two populations in the hand-manipulation-related activity, first we investigated the composition of Best-, Second best- or Worst-grip in SS population by submitting these neurons to the same analysis described above. Five out of 15 SS-related neurons were excluded from further analysis because they did not show any significant main effect for the Grip factor. Figure 5B shows numbers of each grip in the three categories. The results of chi-square test in the cross tabulation showed a significant deviation between expected values and actual measurements (*χ* ^2^ = 17.4, *df = *4, *p*<.01). Furthermore, a residuals analysis showed that the statistically largest proportion was represented by FE in the Best-grip (*n* = 8, adjusted residual = 3.8, *p*<.01), by PG (*n* = 6) in the Second best-grip category (adjusted residual = −2.2, *p*<.05), and by SG (*n* = 6) in the Worst-grip category (adjusted residual = 2.2, *p*<.05).

Figure 5C shows time course and intensity of the activity of neuronal population of each category. A 3×2 repeated measures ANOVA with Grip (PG, FE, SG) and Condition (Light, Dark) as main factors was applied. The analysis showed a significant main effect for Population [F(2,18) = 12.67, *p*<.001] and Epoch [F(1,9) = 37.83, *p*<.0001]. It also showed a significant interaction between factors [F(2,18) = 6.28, *p*<.01]. Activity of three populations was stronger in the post-contact epoch than in the pre-contact one (*p*<.0001). Importantly, in contrast to hand-manipulation-related neurons (see Figure 4C), SS-related population did not show any significantly different activity in pre-contact epoch. Only the post-contact epoch activity of the Best-grip population was statistically stronger than that of both the Second best- and the Worst-grip populations (for both comparisons, *p*<.001).

**Figure 6:**
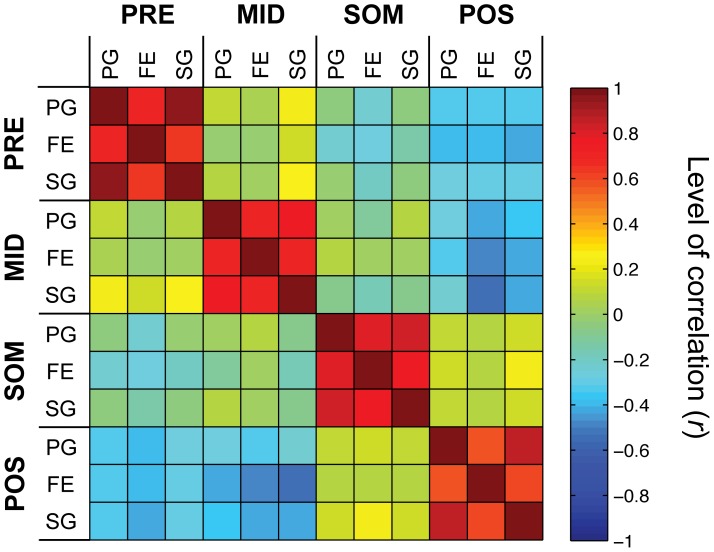
Correlation analysis of hand-manipulation-related activity of among somato-motor populations. Correlation among mean net normalized responses to each of the 3 grips (PG, FE and SG) in the 4 somato-motor types of neurons (PRE, MID, POS and SOM). The correlation map was reordered based on similarity of correlation values among types. Color code indicates correlation value (*r*) between all possible pair of responses.

Since population analysis demonstrated an evident difference both in grip-selectivity and discharge temporal profile between grip-selective hand-manipulation-related neurons and grip-selective SS-related neurons (Figure 4 B, C and Figure 5B, C), we further investigated the relation between the above defined 3 hand-manipulation-related types (PRE, MID, POS; in total, *n* = 33) and SS-related neurons (SOM; *n* = 15). For all 48 neurons we added up the activity both in pre- and post-contact epochs and then calculated the mean values of net normalized activity for each grip (PG, FE and SG). We next evaluated their correlation coefficient (Figure 6). Correlation map showed that each type of neurons (PRE, MID, SOM and POS) showed a significant positive correlation among the 3 grip types (for all pairs, *p*<. 01). Although pairs among PRE, MID and SOM type neurons or pair between SOM and POS ones did not show any significant correlations with others (for all pairs, *p*>. 05, *n.s.*), POS type neurons showed significantly negative correlations with PRE and MID type neurons (for all pairs, *p*<. 05).

### Effect of Visual Feedback during Hand-manipulation

In order to investigate the effect of visual feedback, most of the hand-manipulation-related neurons (*n* = 33) were tested while monkeys performed PG (*n* = 16/33, 48.6%) and/or FE (*n* = 20/33, 60.6%) in the Dark condition.

A 2×3 repeated measures ANOVA with Condition (Light, Dark) and Epoch (baseline, pre-contact, post-contact) was applied. In 16 neurons tested during PG execution in both Light and Dark conditions, 8 neurons (50%) showed statistically stronger response in the dark, 3 neurons (19%) showed the opposite effect, and 5 neurons (31%) did not show any significant difference between the two conditions. On the other hand, in the 20 neurons tested during FE execution in both conditions, 6 neurons (30%) showed statistically stronger response in the dark than in the light, 6 neurons (30%) showed the opposite effect, and 8 neurons (40%) did not show any significant difference between the two conditions.

**Figure 7:**
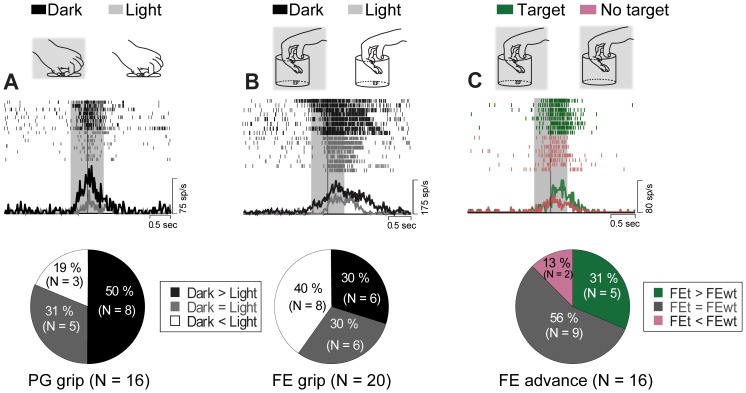
Effect of visual feedback (A-B) and effect of target presence (C) during hand-manipulation. (*A*) Responses in dark (black) and light (gray) conditions during PG execution (*top*). Proportion of statistically defined responses in the two conditions; black indicates neurons showing stronger responses in dark than in light (Dark>Light, *p*<.05); gray indicates Dark<Light (*p*<.05); white indicates Dark = Light (*n.s., p*>.05) (*bottom*). (*B*) Responses in dark (black) and light (gray) conditions during FE execution. Conventions as in (*A*). (*C*) Effect of target presence. Green indicates responses in FE task with a target (FEt); Pink indicates responses in FE task without a target (FEwt). Both conditions were performed in dark (*top*). Proportion of statistically defined responses in two conditions; green indicates neurons showing stronger responses during FEt than FEwt (FEt>FEwt, *p*<.05); brown indicates FEt<FEwt(*p*<.05); gray indicates FEt = FEwt (*n.s., p*>.05).

Figure 7A shows an example of dark selective neuron during PG execution. The ANOVA analysis showed a significant main effect for the factor Condition [F(1, 7) = 208.86, *p*<.0001], Epoch [F(2, 14) = 59.45, *p*<.0001] and also interaction between factors [F(2, 14) = 22.74, *p*<.0001]. Baseline activity was not significantly different between Light and Dark conditions. However, while activity in the pre-contact epoch did not show any significant difference with its baseline in Light condition, that activity in the Dark condition was significantly stronger than during baseline (for each comparison, *p*<.001). Furthermore, activity in post-contact epoch in the Dark condition showed the best response among task-related epochs (for each comparison *p*<.01). Figure 7B shows another example of dark selective neuron tested during FE execution. The analysis showed a significant main effect for the factor Condition [F(1, 14) = 68.67, *p*<.001], Epoch [F(2, 14) = 90.03, *p*<.0001] and interaction between factors [F(2, 14) = 7.20, *p*<.01]. As the previous example, although activity in baseline was not significantly different between conditions, the neuron showed significantly stronger responses in the dark than in the light during both pre- and post-contact epochs (for each comparison, *p*<.005).

### Effect of Target Presence during Hand-manipulation

To examine how much hand-manipulation-related neurons in SII/pIC hand region do show somato-motor context-dependent activity, we analyzed 16 neurons in two different conditions: a) FE grip with a target (FEt), and b) the same grip performed without a target (FEwt). Monkey performed both conditions in the dark (Figure S3). The results of a 2×3 repeated measures ANOVA with Condition (FEt, FEwt) and Epoch (baseline, pre-contact and post-contact) as main factors showed that out of the 16 tested neurons, 5 neurons (31%) showed significantly stronger activity during FEt, 2 neurons (13%) showed significantly stronger activity during FEwt. The remaining 9 neurons (56%) did not show any significant difference between the two conditions.

Figure 7C shows an example of neuron showing significantly stronger responses during FEt execution. The analysis showed a significant main effect for the factor Condition [F(1, 7) = 21.00, *p*<.005], Epoch [F(2, 14) = 78.76, *p*<.0001] and also interaction between factors [F(2, 14) = 10.85, *p*<.0002]. Importantly, the pre-contact epoch activity was not statistically different between FEt and FEwt (*p*<.0001), while activity in post-contact epoch was significantly stronger in FEt than in FEwt condition (*p*<.0001). This result may suggest that the additive discharge after contact in FEt condition could be partly due to an object-displacement effect.

## Discussion

Converging findings from non-human primates and humans brain studies suggested that secondary somatosensory cortex (SII) and the adjacent posterior insular cortex (pIC) could play a pivotal role in somato-motor haptic processing. Although hodological studies in macaque monkey brain revealed that hand regions within SII/pIC have reciprocal connections with the parieto-premotor grasping-related areas, such as ventral premotor area F5 and anterior intraparietal area AIP, the physiological somato-motor properties of SII/pIC during hand-manipulation are still largely unknown.

The original aim of this study was to determine whether neurons in SII/pIC hand regions show hand-manipulation-related properties. We formally tested a total of 48 single units from SII/pIC in two macaque monkeys. The main results of our study can be summarized as follows. (1) Focusing on motor responses, 70% (*n* = 33/48) of task related recorded neurons were only activated during monkeys’ active hand-manipulation. Of those 33 neurons, 15 (45%) became active before hand-target contact, while the remaining neurons were mostly activated after contact. (2) Thirty-percent (*n* = 15/48) of studied neurons responded to both passive somatosensory stimulation and motor task execution. (3) A consistent percentage of all tested neurons was selectively active during finger exploration (FE) and precision grasping (PG) execution. (4) A subset of hand-manipulation-related neurons increased their discharge when visual feedback was absent. (5) Correlation analysis revealed that all tested neurons in this region could be involved in haptic processing during object grasping and hand-manipulation.

### Functional Role of Hand-manipulation-related Neurons

Previous neurophysiological studies of area SII have focused on the discharge patterns of cutaneous mechanoreceptive afferents when tactile stimuli were passively applied onto the glabrous skin of the primate hands [46], [53]–[57]. Although these studies implied the presence of neurons only activated during active hand-manipulation execution without clear somatosensory RFs [18], [19], [58], those were never studied by using active hand-manipulation tasks. Taylor and co-workers [59] proposed two distinct functional comparators in their haptics model; 1) Texture analyzer and 2) Movement analyzer. In their model, they emphasized the importance of motor properties in haptics processing, enabling tactile object/texture recognition. In particular, the importance of efference copy for haptics.

We have focused on the motor aspect of haptics model by testing single unit activity in SII/pIC of macaque monkeys. The present study for the first time demonstrates motor responses in the SII/pIC hand region specifically during different types of hand-manipulation. These type of neurons did not show any passive somatosensory responses on the hand and fingers but only showed a vivid discharge when monkeys grasped/explored a target (Figure 2A–D, Figure 3A). Furthermore, we revealed that hand-manipulation neurons consisted of three independent motor types, Pre-contact (PRE), Middle (MID) and Post-contact (POS) type (Figure 3B, C). We also found that most of them showed grip selectivity. In particular, the distribution of grip-preference index values of hand-manipulation-related neurons appear to be similar to that displayed by grasping neurons in ventral premotor area F5 and in inferior parietal areas AIP and PFG [28], [30], [51].

Both PRE and MID type consisted of neurons discharging prior to the hand-target contact. In particular, as epitomized by the neuron shown in Figure 2A, Pre-contact-selective neurons were active approximately 300 msec before hand-target contact and most importantly, their peak of activity occurred before hand-target contact. On the basis of this evidence, one could speculate that this pre-contact activity might reflect either the direct or the indirect influence of efference copy of selected motor command [15], [28], [60]–[65]. As these neurons, also the MID type ones increased their firing rates before hand-target contact (Figure 2B). However, in contrast to PRE-type, their peak of activity occurred at contact time. Moreover, their discharge lasted in post-contact epoch, when grasp was completed and started the object lifting (Figure 2B). Additionally, the activity of the MID type neurons during both pre- and post-contact epochs was positively correlated in each grip type (Figure 3C, MID). On the basis of this evidence, one could speculate that MID type neurons might be involved in the predicted sensory consequences during hand-manipulation [63], [66]–[69]. In contrast to PRE and MID type neurons, POS type neurons significantly increased their activity during active hand-manipulation right after the onset of hand-target contact (Figure 2C). Since POS type neurons, as the other two types, neither responded to passive proprioceptive nor to tactile stimuli, their discharge likely underpinned active finger movements during target exploration. In fact, the 16 neurons tested in the FE advance task showed significantly stronger motor-related activity than baseline in both FEt and FEwt conditions (Figure 7C). Since the monkey explored inside the cup in both conditions, we interpreted a consistent number of neurons (FEt>FEwt, *n* = 5 and FEt = FEwt, *n* = 9) as showing the effect of active touch during task execution. On the basis of this interpretation, the significant activity enhancement in the FEt task (FEt>FEwt) likely reflects the presence of the target and the following target displacement movements [62], [70]–[72]. In favor of this interpretation, correlation analysis clearly revealed specificity of FE responses in POS type neurons, namely they did not show any significant correlation with other types of grip (Figure 3C).

### Grip-selectivity and Discharge Temporal Profile of Somatosensory and Motor Neurons in SII/pIC

The small number of SS-related neurons was due to our strict criterion to select neurons to be recorded. In fact, since the present study focused on purely motor responses of SII/pIC neurons, we only recorded neurons if we observed stronger responses during active hand-manipulation than during passive somatosensory stimulations. Additionally, we did not test neurons by means of the motor task if they showed strong baseline activity during the set period. In contrast to our study, Gardner and co-workers demonstrated grasping-related activity of somatosensory neurons in SI and posterior parietal cortex, recording neurons with high baseline activity even before motor execution. In our study the baseline activity of SS-related neurons was as low as that of hand-manipulation-related ones (Figure 2E).

Both hand-manipulation-related and SS-related neurons showed grip-selectivity (Figures 5 and 7). On the basis of the comparison between hand-manipulation-related and SS-related neurons, we suggested a specificity of the former: 1) Best-grip type consisted of FE and PG, while Worst-grip was SG; 2) Independently from the grip-selectivity category (Best, Second best and Worst), neurons began to be active before hand-target contact; 3) Activity of Best-grip population was significantly stronger than Worst-grip population in both pre- and post-contact epochs. We suggest that the hand-manipulation-related neurons in SII/pIC code different hand/finger movement strategies depending on task conditions.

On the other hand, activity of SS-related neurons may correlate with duration of hand-manipulation time (Figures 5 B and S2): Thus, 1) Best-grip type was FE (358±131 msec), Second best-grip was PG (276±130 msec), Worst-grip was SG (159±97 msec); 2) Independently of the grip-selectivity category, SS-related neurons were active after hand-target contact; 3) Activity of Best-grip population was significantly stronger than Worst-grip population only during post-contact epoch. We suggest that the SS-related neurons in SII/pIC detect the timing of the hand-object interaction. This may trigger proper motor command for exploring and manipulation of the objects.

### Effect of Visual Feedback in SII/pIC hand-manipulation-related Neurons

Naturally, exploring an object in the dark without any visual feedback is much harder than in the light. Our behavioral analysis showed that the hand-manipulation execution time in the Dark was longer than in the Light condition (Figure S2). Moreover, a consistent percentage of neurons fired stronger in the Dark than in Light condition (Figure 7A, B). These responses might be related to monkey’s attentive focus during object exploration [42], [44], [45], [73]. Importantly, motor responses started approximately 300–400 msec before hand-target contact. This link between the preparation of goal directed hand-manipulation and attention is consistent with the premotor theory of attention [74]–[76]. Such theory suggested that common mechanisms are involved in the control of both action and attention and it holds that attentional shifts are triggered whenever sensory-motor brain regions are activated during movement preparation. SII/pIC hand region might be involved in this attentional mechanism binding somato-motor information during specific hand-manipulations. Interestingly, a patient suffering of tactile apraxia reported by Valenza and co-workers [10] because of a lesion of parieto-frontal operculum including SII but not SI, demonstrated severe impairment of tactile object recognition during haptics exploration. Such deficit could be due to a loss of sensory-motor binding function generated by neurons similar to those described in our paper.

It is well known that reward system including SII and insular cortices is activated by eating and food anticipation [77]. Although the aim of our monkeys was to retrieve a food morsel during the motor task, hand-manipulation-related activity did not simply reflect the presence of reward [78], [79]. In the grip-selectivity analyses, both hand-manipulation- and SS-related neurons showed significantly different discharge intensity between Best- and Worst-grip (Figures 4C and 5C). This difference cannot be explained by the presence of reward or by reward-expectancy.

### Conclusion

The present findings suggest that both hand-manipulation-related and SS-related neuronal populations likely contribute to haptics processing from the initial to the final phase of grasping and object manipulation (Figure 6).

As mentioned above, hodological studies show strong reciprocal connections between ventral premotor cortex (PMv) and the hand region of SII/pIC. Given this evidence, we posit that the activity of both PRE and MID type neurons might likely reflect efference copy or corollary discharge of selected motor commands from PMv. When a movement is made, an efference copy of the motor command is used to make a prediction of the sensory consequences of the movement. This sensory prediction can then be compared with the actual sensory feedback or kinesthesia during movement used to optimize motor control.

We also posit that post-contact dominant activity of both SS- and POS-type neurons might likely be involved in hand-object contact detection and hand-finger exploration after the contact respectively. Hand-object contact signals the end of the reaching movement. It provides an important tactile cue for the timing of the hand-object interaction sequence [80]–[82] and corrects current or memorized motor commands for dexterous hand-manipulation[83], [84] [83], [84]. Gardner and co-workers [63], [85], [86] demonstrated that SI (mainly areas 3b and 1) shows larger number of these two types of neurons than posterior parietal cortex (PPC, mainly areas 5 and 7a). One could speculate that SII/pIC SS- and POS-type neurons collaborate with SI neurons in signaling hand-object contact. Furthermore, hand-finger exploration brings objects into the best configuration to be grasped, through their rotation or sliding before finally grasping them. The object depending exploratory strategy could provide haptic perception of the shape [87], [88].

Unlike SI and superior parietal cortex (areas 2 and 5), the SII/pIC region shows dense reciprocal connections with the ventrolateral prefrontal cortex. In particular, both the intermediate area 12r [89] and the rostral part of area 46vc (bank and convexity) [90] show connections with the hand region of SII. Borra and co-workers suggested that although the possible contribution of the intermediate area 12r to hand-manipulation is still unknown, it might contribute to the non-spatial memory information related to object physical properties such as weight and texture. On the other hand, Gerbella and co-workers suggested that the rostral part of area 46vc might be involved in selecting, monitoring, and updating object-oriented hand actions depending on behavioral goals (see also [91]). We suggest that object properties based on sensory-motor information could be sent from SII/pIC and PMv areas to the prefrontal cortex to select the adequate motor programs depending on different contexts or task demands [92], [93]. Such motor-related activity could perhaps also provide the somato-motor binding principle enabling the translation of diachronic somatosensory inputs fed by peripheral receptors into a coherent image of the explored object.

## Supporting Information

Figure S1 **Functional mapping of somato-motor properties in SII/pIC.** Unfolded view of the lateral sulcus of both right and left hemispheres of MK1 (*Left*). Example of one coronal section (AP 9) showing the position of anatomical markers (Reference point 1–4; R1–R4) to build the unfolded map (*Right*). The 2D reconstruction of the upper bank of the lateral sulcus (UBLs) and of the posterior insula was aligned along its fundus, indicated by a straight dashed line (Reference point 3; R3). The continuous lines mark the lips of the upper (R1), the starting point of the circular region (R2), and the wall of the middle insular region (R4). Arrows mark the most anterior tip of the intraparietal (IPs) and central sulcus (Cs). Each color dot indicates the entrance point of the electrode. Green dot: Mouth; blue dot: face; red dot: hand/fingers; yellow-green dot: arm; black dot: lower body part; cyan dot: hemi-body; pink dot: whole body somatosensory representation. Red dot within black circle indicates the recording site of hand-manipulation-related neurons. Calibration bar: 10 mm.
(TIF)

Figure S2 **Kinematic analyses in Light and Dark conditions.** (*A*) Maximal finger aperture (cm) during the execution of three different grips both in the Light and Dark conditions. (*B*) Reaching and pre-shaping time (msec) for three grips both in the Light and Dark conditions. (C) Hand-manipulation execution time (msec) for three grips both in Light and Dark conditions. (D) Bringing to the mouth execution time (msec) for three grips both in Light and Dark conditions. For each parameter, error bars indicate ± SEM (standard error of the mean), *p<.001.
(TIF)

Figure S3 **Kinematics analyses in FE advance task.** (*A*) Maximal finger aperture (cm) during the execution of FEt and FEwt. (*B*) Reaching and pre-shaping time (msec) of FEt and FEwt. (*C*) Hand-manipulation execution time (msec) of FEt and FEwt. For each parameter, error bars indicate ± SEM (standard error of the mean), *p<.001.
(TIF)

Text S1 **Text of supporting informaiton for Figure S1.**
(DOC)

Text S2 **Text of supporting information for Figure S2.**
(DOC)

Text S3 **Text of supporting information for Figure S3.**
(DOC)
