# Supplementary figures and images for: Somato-Motor Haptic Processing in Posterior Inner Perisylvian Region (SII/pIC) of the Macaque Monkey
Source: PLoS One. 2013 Jul 30;8(7):e69931. doi: 10.1371/journal.pone.0069931 (PMC3728371; doi:10.1371/journal.pone.0069931)

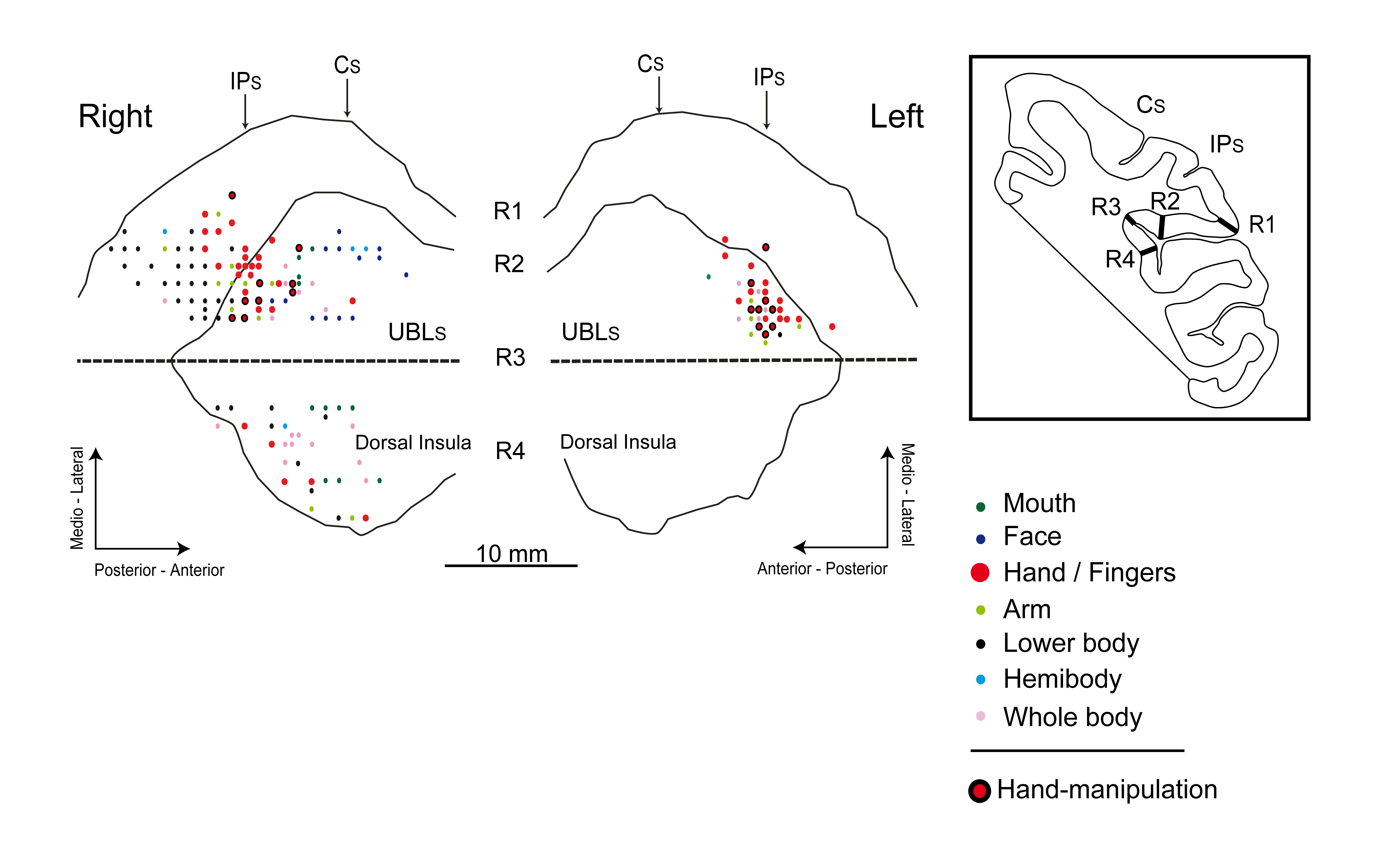

Supplement: Figure S1 — Functional mapping of somato-motor properties in SII/pIC. Unfolded view of the lateral sulcus of both right and left hemispheres of MK1 (Left). Example of one coronal section (AP 9) showing the position of anatomical markers (Reference point 1–4; R1–R4) to build the unfolded map (Right). The 2D reconstruction of the upper bank of the lateral sulcus (UBLs) and of the posterior insula was aligned along its fundus, indicated by a straight dashed line (Reference point 3; R3). The continuous lines mark the lips of the upper (R1), the starting point of the circular region (R2), and the wall of the middle insular region (R4). Arrows mark the most anterior tip of the intraparietal (IPs) and central sulcus (Cs). Each color dot indicates the entrance point of the electrode. Green dot: Mouth; blue dot: face; red dot: hand/fingers; yellow-green dot: arm; black dot: lower body part; cyan dot: hemi-body; pink dot: whole body somatosensory representation. Red dot within black circle indicates the recording site of hand-manipulation-related neurons. Calibration bar: 10 mm. (TIF) [file pone.0069931.s001.tif]

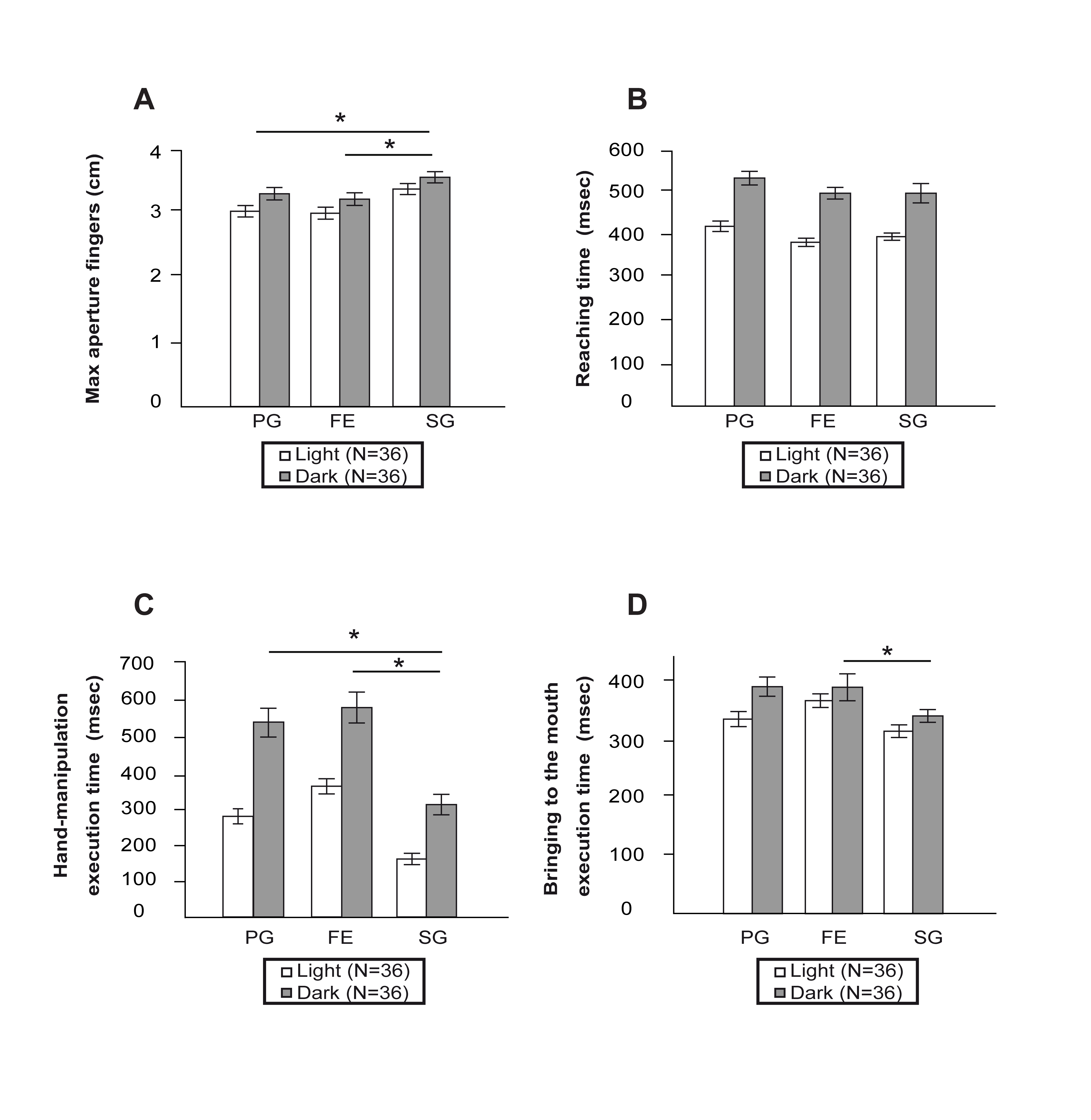

Supplement: Figure S2 — Kinematic analyses in Light and Dark conditions. (A) Maximal finger aperture (cm) during the execution of three different grips both in the Light and Dark conditions. (B) Reaching and pre-shaping time (msec) for three grips both in the Light and Dark conditions. (C) Hand-manipulation execution time (msec) for three grips both in Light and Dark conditions. (D) Bringing to the mouth execution time (msec) for three grips both in Light and Dark conditions. For each parameter, error bars indicate ± SEM (standard error of the mean), *p<.001. (TIF) [file pone.0069931.s002.tif]

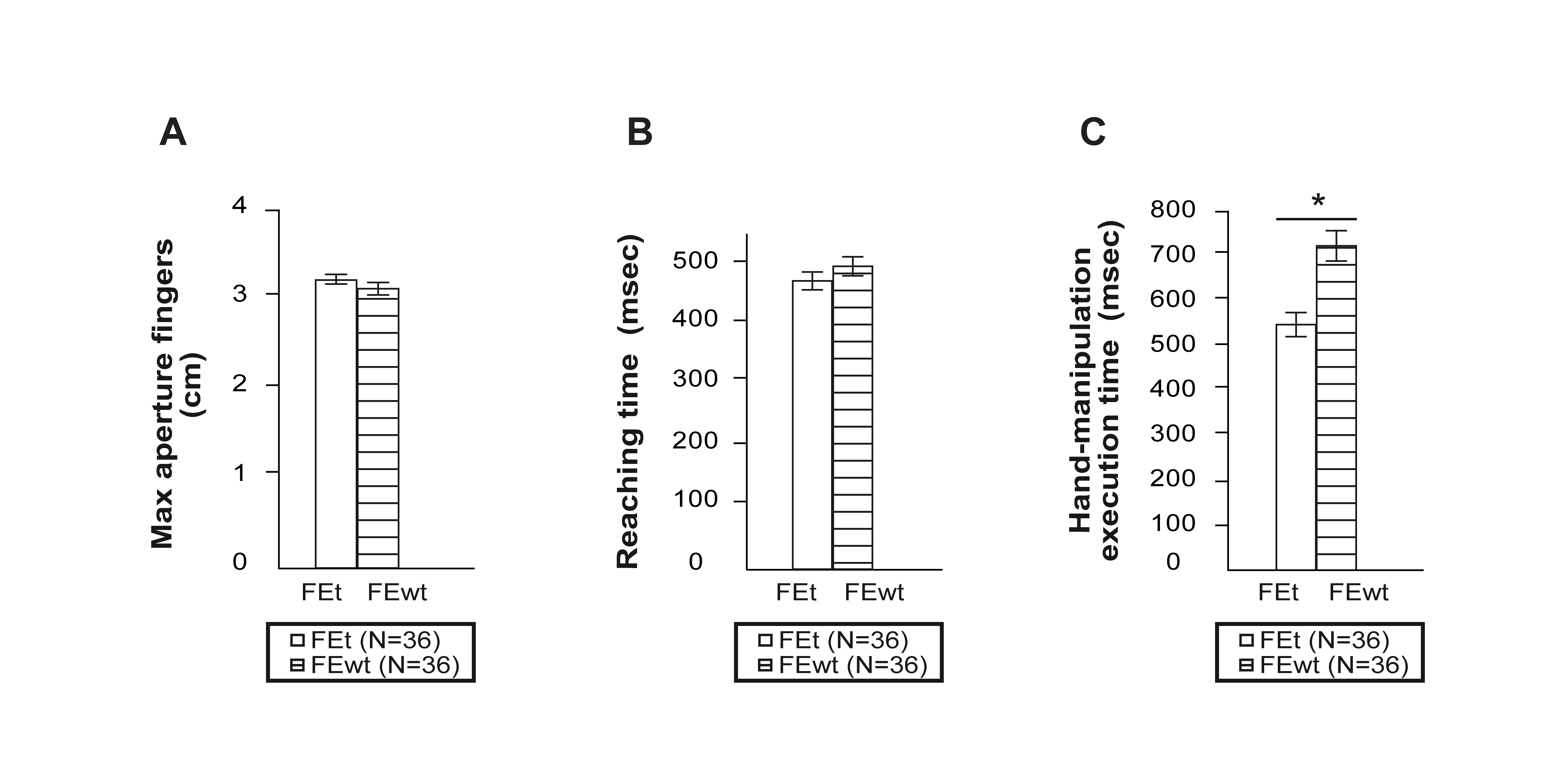

Supplement: Figure S3 — Kinematics analyses in FE advance task. (A) Maximal finger aperture (cm) during the execution of FEt and FEwt. (B) Reaching and pre-shaping time (msec) of FEt and FEwt. (C) Hand-manipulation execution time (msec) of FEt and FEwt. For each parameter, error bars indicate ± SEM (standard error of the mean), *p<.001. (TIF) [file pone.0069931.s003.tif]
