# Supplementary material for: Somato-Motor Haptic Processing in Posterior Inner Perisylvian Region (SII/pIC) of the Macaque Monkey
Source: PLoS One. 2013 Jul 30;8(7):e69931. doi: 10.1371/journal.pone.0069931 (PMC3728371; doi:10.1371/journal.pone.0069931)
Supplement: Text S1 — Text of supporting informaiton for Figure S1. (DOC) [file pone.0069931.s004.doc]

**Functional mapping of somato-motor properties in SII/pIC.**

Figure S1 shows the somatotopy of the posterior inner perisylvian region including area SII and the adjacent region of posterior insular cortex (pIC). The face and oral structures (teeth, gums, palate) were represented in the rostral part, the hand and arm were represented in the middle part, while the foot and leg were represented in the caudal part.

In spite of the lack of agreement on the extension and parcellation of SII hand area and its nomenclature, the location of physiologically defined hand region is robustly consistent among previous findings [1-8]. Krubitzer and colleagues [1] by means of multi units recording on anesthetized monkeys (*Macaca fascicularis*) demonstrated two symmetric body representations in the upper bank of the Sylvian fissure they called SII and PV (parietal ventral area). On the other hand, Fitzgerald and colleagues [3] by recording single unit from awake, behaving monkeys (*Macaca mulatta*) suggested the presence of three hand-finger areas along the rostro-caudal axis, they referred to as the anterior, central and posterior fields. According to these authors, the anterior and posterior fields contain neurons responding to proprioceptive stimuli, while the central field contains neurons responding to cutaneous stimuli. Furthermore, they speculated that the anterior field corresponds to area PV and that the central and posterior fields correspond to Krubitzer’s area SII proper. The hand-finger representation we report in this paper was localized in the medial part of the upper bank of Sylvian fissure, likely at the border between parietal operculum and posterior granular Insula, most likely overlapping with the anterior and central fields described by Fitzgerald et al.[3]. For this reason we refer to this region as SII/pIC.

**References**

1. Krubitzer L, Clarey J, Tweedale R, Elston G, Calford M (1995) A redefinition of somatosensory areas in the lateral sulcus of macaque monkeys. J Neurosci 15: 3821–3839.

2. Pons T, Garraghty P, Mishkin M (1988) Lesion-induced plasticity in the second somatosensory cortex of adult macaques. Proceedings of the National Academy of Sciences 85: 5279–5281.

3. Fitzgerald PJ, Lane JW, Thakur PH, Hsiao SS (2004) Receptive field properties of the macaque second somatosensory cortex: evidence for multiple functional representations. Journal of Neuroscience 24: 11193–11204.

4. Robinson CJ, Burton H (1980) Organization of somatosensory receptive fields in cortical areas 7b, retroinsula, postauditory and granular insula of M. fascicularis. J Comp Neurol 192: 69–92.

5. Schneider RJ, Friedman DP, Mishkin M (1993) A modality-specific somatosensory area within the insula of the rhesus monkey. Brain Res 621: 116–120.

6. Jezzini A, Caruana F, Stoianov I, Gallese V, Rizzolatti G (2012) Functional organization of the insula and inner perisylvian regions. Proceedings of the National Academy of Sciences 109: 10077–10082.

7. Burton H, Fabri M, Alloway K (1995) Cortical areas within the lateral sulcus connected to cutaneous representations in areas 3b and 1: a revised interpretation of the second somatosensory area in macaque monkeys. J Comp Neurol 355: 539–562.

8. Friedman DP, Murray EA, O'Neill JB, Mishkin M (1986) Cortical connections of the somatosensory fields of the lateral sulcus of macaques: evidence for a corticolimbic pathway for touch. J Comp Neurol 252: 323–347.
