# Supplementary material for: Somato-Motor Haptic Processing in Posterior Inner Perisylvian Region (SII/pIC) of the Macaque Monkey
Source: PLoS One. 2013 Jul 30;8(7):e69931. doi: 10.1371/journal.pone.0069931 (PMC3728371; doi:10.1371/journal.pone.0069931)
Supplement: Text S2 — Text of supporting information for Figure S2. (DOC) [file pone.0069931.s005.doc]

**Kinematic analyses in Light and Dark conditions.**

Kinematics analyses were carried out on both monkeys in order to verify the presence of possible differences in the parameters of hand movement when monkeys performed different types of hand-manipulations (PG, FE and SG) in both Light and Dark conditions (Figure S2).

We focused on four parameters: 1) Reaching and pre-shaping, defined as the time from home key release to hand-target contact; 2) Hand maximal aperture, defined as the maximal distance between the tip of thumb and index finger during reaching time; 3) Duration of hand-manipulation execution, which was defined as the duration measured from object contact until the monkey started lifting up the object; 4) Bringing to the mouth, defined as the time from object lifting to object contact with the mouth. In the case of FE condition, since the monkey explored a target in the cup, the tip of the fingers was not detectable, thus hand aperture was calculated before monkey’s fingers entered the cup. White colored markers were placed on the monkey’s tip of the last phalanx of the thumb and index finger. By means of a digital video camera, we captured each hand-manipulation, and analyzed it off-line by means of dedicated software, at a sampling rate of 25 frames/second.

For each parameter, a 3 x 2 repeated measures ANOVA with Grip (PG, FE, SG) and Condition (Light, Dark) as factors followed by Bonferroni post-hoc tests was applied. Concerning reaching time, the analysis showed a significant main effect just for Condition [F(1,35) = 72.23, *p* < .001], but neither a significant effect for Grip nor for the interaction between Grip and Condition. Duration of reaching was longer in the Dark than in the Light conditions (*p < .*0001).

Concerning finger aperture, the analysis showed a significant main effect for the factor Grip [F(2, 70) = 17.01, *p* < .001] and Condition [F(1, 35) = 14.75, *p* < .001], but no interaction between them. Finger aperture was larger during SG than during PG and FE (each comparison, *p < .*0001) in both Light and Dark conditions (*p < .*0001). Importantly, during performance of the same grip, finger aperture was not significantly different between Light and Dark conditions.

Concerning the time of hand-manipulation execution, the same repeated measures ANOVA was applied, revealing a significant main effect for Grip [F(2,70) = 28.82 , *p* < .001] and Condition [F(1,35) = 78.36, *p* < .001], but no interaction between them. Hand-manipulation execution time during SG was shorter than during PG and FE (each comparison, *p < .*0001). In addition, the duration of hand-manipulation execution time in the dark was statistically longer than that performed in the light for the same grip (*p < .*0001).

The analysis of the bringing to the mouth execution time revealed a significant main effect just for Grip [F(2,7s0) = 6.123, *p* < .001], but neither for Condition nor for the interaction between them. Bringing to the mouth execution time during SG was shorter than during PG and FE (each comparison, *p < .*0001). The duration of this movement was longer in the Dark than in the Light condition (*p < .*0001).
