# Supplementary material for: Somato-Motor Haptic Processing in Posterior Inner Perisylvian Region (SII/pIC) of the Macaque Monkey
Source: PLoS One. 2013 Jul 30;8(7):e69931. doi: 10.1371/journal.pone.0069931 (PMC3728371; doi:10.1371/journal.pone.0069931)
Supplement: Text S3 — Text of supporting information for Figure S3. (DOC) [file pone.0069931.s006.doc]

**Kinematics analyses in FE advance task.**

In order to examine the difference between FEt and FEwt, kinematics analyses were carried out (Figure S3).

We captured each trial of both FEt and FEwt by means of a digital video camera, and analyzed it off-line by means of dedicated software, at a sampling rate of 29 frames/second. In both conditions monkey explored a target inside the cup, so the tip of the fingers was not visible after entering the cup. White markers were placed on the monkey’s tip of the last phalanx of the thumb and index finger.

We focused on three parameters: 1) Reaching and pre-shaping, defined as the time between the home key release and fingers’ tip contact with the bottom of the cup. Since finger tips were not visible after entering into the cup, we estimated the contact with the bottom of the cup as the first frame when hand bent because touching the bottom of the cup; 2) Hand maximal aperture, defined as the maximal distance between the tip of thumb and index finger during reaching time; 3) Duration of exploration, defined as the duration measured between the frame related to the estimated contact with the bottom of the cup and beginning of monkey’s hand lifting with or without object, in FEt and FEwt respectively.

For each parameter in both conditions, a two-tail *t*-test was applied. The duration of hand-manipulation execution during FEwt was longer than during FEt (*t* = -3.849; *df* = 70; *p* = .00026). However, both hand maximal aperture and reaching and pre-shaping time before the hand-target/cup contact did not show any significant difference (for each pair *p >*.05).
